# A Stochastic Individual-Based Model of the Progression of Atrial Fibrillation in Individuals and Populations

**DOI:** 10.1371/journal.pone.0152349

**Published:** 2016-04-12

**Authors:** Eugene T. Y. Chang, Yen Ting Lin, Tobias Galla, Richard H. Clayton, Julie Eatock

**Affiliations:** 1 Insigneo Institute for in-silico Medicine and Department of Computer Science, The University of Sheffield, Sheffield S1 4DP, United Kingdom; 2 School of Physics and Astronomy, The University of Manchester, Manchester M13 9PL, United Kingdom; 3 Department of Computer Science, Brunel University London, Uxbridge UB8 3PH, Middlesex, United Kingdom; Gent University, BELGIUM

## Abstract

Models that represent the mechanisms that initiate and sustain atrial fibrillation (AF) in the heart are computationally expensive to simulate and therefore only capture short time scales of a few heart beats. It is therefore difficult to embed biophysical mechanisms into both policy-level disease models, which consider populations of patients over multiple decades, and guidelines that recommend treatment strategies for patients. The aim of this study is to link these modelling paradigms using a stylised population-level model that both represents AF progression over a long time-scale and retains a description of biophysical mechanisms. We develop a non-Markovian binary switching model incorporating three different aspects of AF progression: genetic disposition, disease/age related remodelling, and AF-related remodelling. This approach allows us to simulate individual AF episodes as well as the natural progression of AF in patients over a period of decades. Model parameters are derived, where possible, from the literature, and the model development has highlighted a need for quantitative data that describe the progression of AF in population of patients. The model produces time series data of AF episodes over the lifetimes of simulated patients. These are analysed to quantitatively describe progression of AF in terms of several underlying parameters. Overall, the model has potential to link mechanisms of AF to progression, and to be used as a tool to study clinical markers of AF or as training data for AF classification algorithms.

## Introduction

Atrial fibrillation (AF) is the most prevalent disorder of heart rhythm. It is characterised by an irregular or rapid heartbeat, is increasingly common as patients become older, and is associated with a five-fold increase in the incidence of ischaemic stroke [1]. Strokes are debilitating for the patient and can be very costly for healthcare providers, and so clinical guidelines aim to provide clinicians with the most cost-effective care pathway for AF patients [2, 3]. Much is known about the biophysical mechanisms that initiate and sustain AF in animal hearts [4], but this knowledge is at present not fully taken into account when developing guidance for cost effective treatment at patient and population level.

In this study, we present a stochastic individual-based model that aims to bridge biophysical models and population-level models used in policy making. Our model embeds a stylised representation of biophysical mechanisms of AF, and can be used to simulate the progression of AF in an individual throughout their lifetime. The model has been developed to identify biophysical features that have the greatest impact on cost-effectiveness at a population level, and ultimately could be developed to influence the guidance provided to clinicians. The model presented in this paper is a first step in this process and our aim is to open debate about the most influential features and and how they should be represented. The output of the model is a time series of AF episodes in an individual over a time span of decades, which captures progression starting from short intermittent episodes of AF that gradually become longer until the patient is either in a constant state of AF or dies. The potential applications that a model like this could contribute to include: (i) Predicting or investigating the impact that different treatment strategies (e.g. rate/rhythm control, ablation) on the progression of AF in an individual; (ii) understanding the drivers of progression, that could be exploited by new therapeutic interventions; and (iii) measuring and quantifying progression in individuals to facilitate remote monitoring and tele-health applications.

## Mechanisms and progression of AF

In AF, regular beats arising in the natural pacemaker of the heart are suppressed by rapid and self-sustaining electrical activity. The transition from sinus rhythm (SR) to AF is characterised by a combination of a triggering event and vulnerability of the atria to such triggers [5]. This combination results in self-sustaining rotating waves of electrical activation. Examples of triggering events are a rapid increase of the atrial rate resulting from altered autonomic balance, rapid firing of a high frequency source, and ectopic electrical beats within the atria occurring at a critical time during atrial repolarisation [6, 7]. Increased vulnerability or triggers are associated with structural abnormalities in the atria such as fibrosis and hypertrophy [8, 9], as well as morphological or electrical changes such as gap junctional lateralisation [10] or altered electrical behaviour of cardiac cells [4, 5, 11–14]. The increased rate of excitation and recovery associated with episodes of AF acts to increase vulnerability to further episodes, a process described as AF-induced remodelling [11].

Three stages have been broadly defined in the clinical progression of AF: (i) *paroxysmal AF*, (ii) *persistent AF*, and (iii) *permanent AF* [1]. In *paroxysmal AF*, a AF episode has a typical duration of minutes or hours, lasting up to 7 days, with episodes spontaneously terminating. Inter-episode times can vary greatly from days to months [15], and they will often become shorter as the condition progresses, due to physiological remodelling [11, 14, 16]. The next stage, *persistent AF*, is indicated when the patient has AF episodes lasting 7 days or more, which do not self terminate and require medical intervention such as cardioversion. A patient is deemed to be in *permanent AF* typically when interventions to terminate AF have failed, and the patient is in a near-constant state of AF. An additional classification of *longstanding persistent AF* is often used clinically to identify those patients whose episodes have continued for more than 1 year, although this term is not used within the NICE (National Institute for Health and Care Excellence) clinical guidelines [2, 3]. This classification is based on consensus for simplicity and on clinical relevance [17]. In practice it is difficult to accurately determine a patient’s status in this classification, as long-term continuous monitoring and detailed information about the exact times of initiation and termination of AF episodes would be required. This means that in some instances it can appear that some people revert to a previous stage of AF [18], although European Guidelines [1] technically do not allow for this within their classifications.

## Models of AF

The mechanisms and progression of AF have been extensively studied, and many models have been developed covering a range of granularity from cardiac cells, tissues, organs, individuals through to populations. In general, models of cell, tissue and organ electrophysiology have been used together with experimental work to gain mechanistic understanding, while models of individuals and populations have been used to assess the societal impact and budget planning for health services.

### Mechanistic and biophysical models

One of the earliest computational models of AF represented atrial tissue as a cellular automaton [19], and although this was a very simple model, it captured many features now known to be important in AF, including rotating re-entrant waves. More recent models capture the detailed biophysical behaviour of atrial cells and tissue, including the effects of remodelling on the cellular action potential [20], the autonomic nervous system [21], anatomical detail [22], and structural changes including fibrosis [23].

These models have been used to show that the AF can be initiated by a combination of trigger and vulnerability, and to demonstrate that AF-induced remodelling of cells and tissues both increases vulnerability to further episodes and reduces the likelihood of spontaneous termination [24]. However, these models are computationally intensive to run, and so they are impractical for studies of AF dynamics over time scales of more than a few heartbeats and seconds.

### Individual progression

Most research at an individual level has been associated with predicting the likelihood of a patient experiencing an adverse effect such as ischaemic stroke. Consequently, point-based algorithms based on retrospective studies of large trials such as the Framingham Heart study, and the Euro Heart Survey of AF, have been developed to assess stroke risks and bleeding risks in AF patients. The CHADS_2_, CHA_2_DS_2_-VASc, and HAS-BLED algorithms are based on a patient’s co-morbidities (e.g. cardiovascular disease, hypertension, age, diabetes), and have been validated in several studies (see [25] for an example). A similar algorithm for predicting progression of AF is the HATCH score [26], which has proved to be a “modest predictor of progression” [27]. The possible reasons for these modest predictions are outlined by Jahangir and Murarka [28], but the main criticisms of the HATCH algorithm are first the short follow-up period of 1 year, and second that the initial study on which the algorithm is based included patients from 35 different European countries with different management practices.

Presumably for simplicity and ease of use in a clinical setting, the HATCH algorithm does not include any biophysical information which may be relevant in predicting progression, such as AF-induced remodelling as a result of the proportion of recent time spent in AF (AF burden), and this may contribute to its lack of predictive power. Regardless of these potential deficiencies, the HATCH score still performs better as a predictor of progression than any other independent predictor [26]. However, the HATCH score was developed to predict progression at 1 year rather than long-term progression, and many patients live with asymptomatic AF for many years before it progresses to persistent AF. For such patients the HATCH score is hence of limited scope.

### Policy-level models

In the UK, NICE is the body responsible for making decisions that are consistent with maximizing population health gains subject to budget constraints of the NHS (National Health Service) [29]. Health economists conduct cost-effectiveness analyses using decision models to estimate the long-term costs and health outcomes associated with alternative treatment options for defined patient groups [30]. These are often measured in QALY (quality-adjusted life years) gains per monetary unit spent. Usually such studies at population level take the form of aggregate (cohort) models, for example decision trees or state-transition Markov models drawing data from randomised control trials or health technology assessments if and where available.

In these types of model, time advances in discrete steps, and at each time step patients move to the next appropriate state, based on transition probabilities. As all patients are considered for status updates synchronously, costs and QALYs can be easily calculated and accumulated over a given time horizon. The results from these models then form the basis of the clinical guidelines which aim to assist clinicians in making appropriate and cost-effective treatment decisions. For pragmatic reasons, these models tend not to include detailed individual patient characteristics, and therefore transition probabilities are based on (sub-) population averages. This indicates that individual characteristics of a patient (which may affect the effectiveness of the treatment for this particular patient) may be not considered when assigning to a treatment strategy. The MAPguide project [31] attempted to overcome this by using individual-level discrete-event simulation models to inform policy decisions, but found the level of detail and time required to build such models was prohibitive within the time scale of guideline development.

## Conceptual description of AF progression model

Our aim in this study is to link these modelling paradigms in a stylised population-level model, in which we capture the natural progression of AF in an individual taking into account some of the physiological changes underlying progression.

The scope of such a stylised model is not to make quantitative predictions directly. The purpose is to identify and discuss some of the fundamental mechanisms at work, and to detect underlying principles at intermediate scale. Putting these factors into a mathematical model provides a framework for further thinking. Often initial ideas for phenomena arise at qualitative level (in the context of AF for example this could be AF-induced remodelling, i.e. statements such as “AF begets AF”). Trying to quantify these effects, even in the most stylised manner, sharpens the discussion, and helps to ask the right questions. An initial model can be of use, even if it is fairly basic, for example because it does not capture all relevant factors or because it is not parametrized and validated against statistical data. Even if it later turns out that the initial model is not fully representative, a null model can raise the questions needed to sharpen the understanding of the interplay of mechanistic processes at short time scales, and AF progression in the long-term. Ultimately this may then lead to the construction of more realistic and detailed models, bridging the gap between mechanistic models and models of AF progression in later steps.

Such stylised approaches have successfully been used for example in epidemics (e.g. in the context of the SIR model (susceptible-infective-recovered) [32], cancer modelling (branching processes, evolutionary dynamics, see e.g. [33] for a recent review), or in developmental biology and gene regulation [34]. In the latter context stylised models have for example been used to identify transcriptional and translational delays as crucial for oscillatory behaviour, this was then confirmed in real-world genetic systems [35].

**Fig 1:**
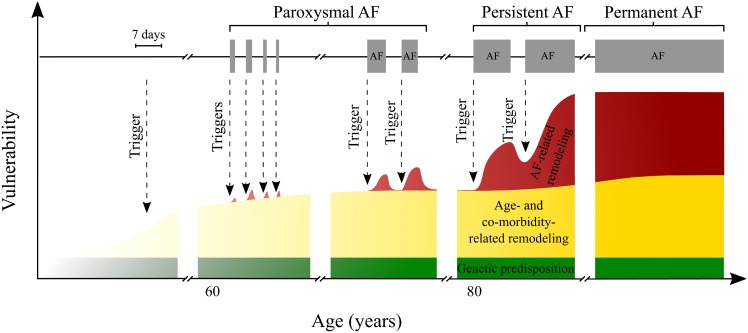
A framework of AF initiation, maintenance and progression, based on [36]. AF onset is dependent on substrate vulnerability and triggers, as described by Heijman et al [36]. Within the present study vulnerability and triggers are combined in to a single factor—activation rate. Activation rate is then dependent on a constant genetic predisposition, time-varying age/co-morbidity-related remodelling, AF-induced remodelling dependent on AF history and trigger events. Physiological changes may increase both the rate of a trigger event occurring and likelihood of AF episode initiation following a trigger event. Over time, some patients progress on to paroxysmal, persistent and permanent AF as their substrate vulnerability and frequency of trigger events increases. Note the timescale for AF triggers and episodes is distinct from the lower axis and is expanded for clarity.

The starting point for the general concept of our model are the factors contributing to AF progression as shown in Fig 1, which is based on [36]. The approach incorporates three different aspects of AF progression: genetic disposition, disease/age related remodelling, and AF-related remodelling. Each of these contribute to the rates with which episodes of AF both begin and end. Our model encodes and quantifies the conceptual model shown in this figure.

The key processes described by our model are initiation and termination of episodes of AF, and we ignore external interventions such as pacemakers, cardioversion, or effects of medication. The likelihood of initiation and termination of AF in the model is dependent upon remodelling of structure and function in atrial tissue, which occur over multiple time scales [37–39].

In our stylised model the atrial rhythm of a patient is described by a binary state: the patient can either be in AF or SR. We adopt a coarse-grained picture of AF initiation, where we describe the activation rate due to both increased triggering events and vulnerability of the atrial substrate. Changes in patient-specific behaviour or physiology over their lifetime will affect both the frequency of triggering events [40, 41] and the vulnerability of the atrial substrate [41]. We do not specifically dissect the relative contribution of physiological changes to substrate vulnerability or frequency of triggers, or model these explicitly. Our model is in terms of an activation rate for episodes of AF, and this rate reflects the combination of triggering events and vulnerability to such triggers.

We consider three factors contributing to a patient’s activation rate: (i) a genetic predisposition, (ii) an age- and co-morbidity related factor, and (iii) previous history of the patient. We consider genetic pre-disposition to be constant and independent of age. Activation related to age and co-morbidities increases with time; we assume implicit correlation of age to other co-morbidities (for example hypertension and hypertrophy), and therefore we do not consider independent effects of specific co-morbidities in the present study. The third element—previous history of the patient—represents the effect of electrical and structural remodelling arising from previous episodes of AF. The activation rate of a patient at any one time depends on the time spent in AF previously and on the number of episodes the individual has experienced so far, and when these occurred.

Factors (ii) and (iii) capture the effects of physiological remodelling: Structural and electrical properties of the atria are modulated by risk factors including ageing and previous history of AF [4, 5]. All factors are patient-specific: different patients in the simulation can, in principle, have distinct parameter settings.

The biophysical mechanisms leading to spontaneous termination of an episode of AF are much less studied and understood than those leading to initiation. Our formulation is based on the hypothesis that the factors contributing to the rate with which episodes terminate are similar to those contributing to activation. Any factor leading to an increase in the initiation rate is taken to cause a reduction in the termination rate. Specifically, we assume one contributing factor reflecting age and co-morbidities. This term in the recovery rate decays (exponentially) as the patient ages, and does not depend on previous history of AF progression. A second component depends on previous history: the propensity to recover from an episode of AF is reduced by time spent previously in the AF state.

Recurrence of an AF episode shortly after an existing episode has been observed in clinical studies [42]. Kaemmerer et al [43] suggest that the likelihood of recurrence is highest immediately after termination and decreases over time, and that a long episode is likely to be followed by multiple shorter episodes immediately after. In order to capture this effect we assume that activation rate (probability of transition to AF per unit time) is increased immediately after an episode and exponentially decays to baseline, which is independent of AF history. We assume the recovery rate (probability of transition from AF to SR per unit time) follows a similar trend; however, the history-based component of AF recovery rate transiently decreases with increasing time spent in an episode. To capture the clustering effect (long episodes are immediately followed by a number of short episodes) we assume the recovery rate overshoots above baseline immediately after an episode terminates, then decays back to baseline.

## Mathematical description of model

Our model describes a patient as either in AF or in SR at any one time. A patient’s trajectory is therefore a series of points in time at which AF episodes begin and end. The starting point of the simulation is the birth of the patient. An important feature of the model is that all episodes of AF are available in the simulation. This is different to the clinical setting, where not all episodes of AF are detected. A real patient would not necessarily be diagnosed to have paroxysmal AF upon initial occurrence of an AF episode. Thus we use the following classification in our simulations: (i) A patient is considered to progress to paroxysmal AF immediately following an episode of at least 14.4 minutes (1% of a day); (ii) they are said to be in persistent AF following an episode lasting 7 days; and (iii) they progress to permanent AF from the point in time at which they enter an AF episode which continues until the end of the simulation at age 100 years, without return to sinus rhythm.

Our model describes the trajectory of individual patients throughout their life time. Patients can be simulated individually, and they do not interact. Time in the model is continuous, and at each point in time *t* a patient is characterised by three attributes, their AF status *S*(*t*), their activation rate, *A*(*t*), and their recovery rate *R*(*t*).

The AF status indicates if the patient is in AF at time *t*, or in SR; if they are in AF we write *S*(*t*) = 1, otherwise *S*(*t*) = 0. As far as the occurrence of AF episodes is concerned a patient trajectory is fully characterised by the values *S*(*t*) for all times *t*. Simulations start at age of zero years, this is labelled *t* = 0. They end at age 100 years (*t* = 100).

The behaviour of *S*(*t*) is determined by the activation and termination rates of AF episodes. Suppose the triggering events of the patient occurs with a rate *f*(*t*), and the vulnerability—defined as a conditional probability of a triggering event successfully induces an AF episode—of the patient is *V*(*t*). Then, if a patient is in sinus rhythm at time *t* they can begin a new AF episode with an activation rate *A*(*t*) = *f*(*t*)*V*(*t*) in our simulation. Both the trigger frequency and vulnerability of the substrate are thought to be dynamic processes, affected by multiple mechanisms and potentially self reinforcing. Moreover, it is unclear whether specific physiological mechanisms modulate just one of *f*(*t*) or *V*(*t*), or both. Thus, whilst these processes are explicitly stated here, the formulation of *A*(*t*), combining both *f*(*t*) and *V*(*t*) in to a single random process, was chosen for this study, leaving the capacity for *f*(*t*) and *V*(*t*) to be investigated in detail in future studies. Conversely, if a patient is in an episode of AF at time *t* (*S*(*t*) = 1) they may return to sinus rhythm with a rate *R*(*t*).

Formally the model describes a binary process, in which *S*(*t*) flips between the two states *S* = 0 and *S* = 1 at random times. The statistics of these transitions are governed by the time-dependent rates *A*(*t*) and *R*(*t*), both of which have units of inverse years. The underlying biophysical elements of AF initiation and termination we wish to describe are encapsulated in the detailed mathematical forms of these rates. It is important to keep in mind that these depend on the prior history of the patient, and so *A*(*t*) and *R*(*t*) are not set quantities, but random variables themselves. We will describe the mathematical forms of these rates next. In doing this we will first define the general mathematics, and not specify the numerical values of the various model parameters we are about to introduce. These will be discussed further below.

### Activation rate *A*(*t*)

Activation rate *A*(*t*) is made up of three components: genetic predisposition, age and co-morbidity related activation, and episode-induced activation [36]. We write this as

$$A\left(t\right)=A_{0}+A_{\text{age}}\left(t\right)+A_{\text{epi}}\left(t\right).$$ (1)

The quantity *A*_0_ is the activation associated with genetic predisposition, it is constant in time, and may vary from patient to patient. The second component, *A*_age_(*t*), is the activation due to the triggering rate and substrate vulnerability associated with age and co-morbidities, both of which are thought to increase over time. In the present study we do not model co-morbidities independently or explicitly, and simply assume that *A*_age_(*t*) depends primarily on age (i.e. other co-morbidities are implicitly correlated with age), but not on the prior AF-history of the subject. This type of vulnerability increases with time. More specifically we assume a sigmoidal function for the functional form of *A*_age_(*t*),

$$A_{\text{age}}\left(t\right)=\frac{A_{1}}{1+\exp\left(-\frac{t-t_{c}}{t_{d}}\right)}.$$ (2)

Age/co-morbidity-dependent activation rate is low in the initial phases of a subject’s trajectory (i.e. *t* ≈ 0), and it then increases to plateau, *A*_1_, at higher ages. The parameter *t*_*c*_ describes the location of the turning point in this evolution, and *t*_*d*_ characterises the width of the sigmoid, i.e. the duration of the transition between low activation rate and the final plateau.

It remains to define *A*_epi_(*t*) as the activation rate associated with acute AF-induced remodelling, which may cause an increase in both cellular triggers and substrate vulnerability.

The following piecewise process is adopted to model *A*_epi_(t). When the patient is not in AF, i.e. at times when *S*(*t*) = 0, we assume that *A*_epi_ decays exponentially with rate *β*,

$$\frac{d}{dt}A_{\text{epi}}\left(t\right)=-\beta A_{\text{epi}}\left(t\right),\;\text{when}\;S\left(t\right)=0.$$ (3)

When a patient is in AF (i.e., when *S*(*t*) = 1), we assume that the activation rate grows exponentially in time limited by a maximum activation rate of *A*_max_. Mathematically we write

$$\frac{d}{dt}A_{\text{epi}}\left(t\right)=\alpha [A_{\text{max}}-A_{\text{epi}}\left(t\right)],\;\text{when}\;S\left(t\right)=1.$$ (4)

The model parameters *α* and *β* are the relaxation rates of *A*_epi_ back to 0 when *S*(*t*) = 0, or to maximum value when *S*(*t*) = 1.

**Fig 2:**
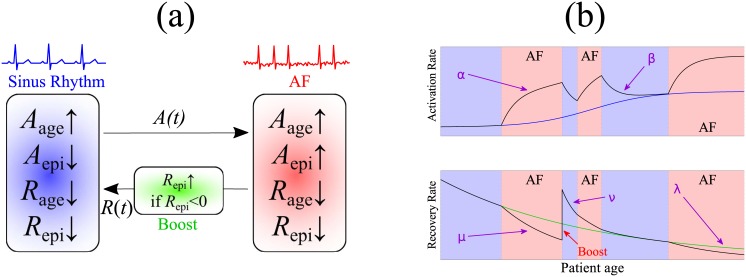
Illustration of the main mechanics of the model. (a) Main elements governing the switching process between sinus rhythm and AF. (b) Time courses of activation rate and recovery rate during AF episodes and in sinus rhythm. activation rate of AF due to ageing, *A*_age_, increases according to a sigmoid function, and age-based recovery rate *R*_age_ decreases exponentially. A healthy patient triggers an AF episode with rate *A*(*t*) and terminates an AF episode with rate *R*(*t*). In AF, episode-based remodelling *A*_epi_ transiently increases activation rate at a rate *α* and decreases the total recovery rate *R* at rate *μ*. When an episode terminates, the recovery rate ‘boosts’ above baseline by a factor of *B*. Back in sinus rhythm, *A*_epi_ decays to zero with rate *β* and *R*_*epi*_ decays with rate *ν* back to the age-based baseline *R*_age_.

We further assert that the activation rate *A*(*t*) is continuous: after a random switching event into AF or out of AF, the initial condition of *A*_epi_(*t*) in the following segment is set as the final value of *A*_epi_(*t*) right before the switching event. A schematic diagram of the evolution of *A*(*t*) is shown in Fig 2.

### Recovery rate *R*(*t*)

Next, we define the recovery rate *R*(*t*) to consist of two components:

$$R\left(t\right)=R_{\text{age}}\left(t\right)+R_{\text{epi}}\left(t\right).$$ (5)

The variable *R*_age_(*t*) stands for a naturally declining ability to recover from an established episode. The decline is due to age, and it is taken to be exponential with rate *λ*,

$$R_{\text{age}}\left(t\right):=R_{0}e^{-\lambda t}.$$ (6)

The second component, *R*_epi_(*t*), is the history-dependent recovery rate, which is known to be a function of preceding episodes of AF [11]. Similar to the above procedure for the activation rate we define a piecewise process for *R*_epi_. When the subject is not in AF, we assume

$$\frac{d}{dt}R_{\text{epi}}\left(t\right)=-\nu R_{\text{epi}}\left(t\right)\;\text{when}\;S\left(t\right)=0,$$ (7)

which describes the recovery from future episodes of AF, and indicates an exponential decay of *R*_epi_ with rate *ν* during times the patient is in SR.

When the patient is in the AF state (*S*(*t*) = 1) we assume that the *total* recovery rate, *R*(*t*) = *R*_age_(*t*) + *R*_epi_(*t*) falls exponentially with rate *μ*

$$\frac{d}{dt}R\left(t\right)=-\mu R\left(t\right),\;\text{when}\;S\left(t\right)=1.$$ (8)

This formulation aims to capture the short-term AF episode-induced remodelling, and acts to reduce the likelihood of termination. Crucially we assume *μ* > *λ*, i.e. the drop in total recovery rate during AF is quicker than the drop of *R*_age_(*t*) when the subject is in sinus rhythm. Typically the total recovery rate *R*(*t*) will fall below the baseline rate *R*_age_(*t*), hence *R*_epi_(*t*) = *R*(*t*) − *R*_age_(*t*) formally turns negative; we comment on this below.

In order to capture the clustering of several short episodes frequently observed clinically after a long AF episode, we introduce an additional ‘boost’ term into the dynamics of the recovery rate. Specifically we use the following procedure when an episode of AF terminates. Say the termination time is *t*. Prior to this moment the patient will have been in AF, and so *R*(*t*) is governed by Eq (8). The age-related contribution is *R*_age_(*t*) = *R*_0_ *e*^−*λt*^, see Eq (6). Together these equations define *R*_epi_ = *R* − *R*_age_ just before the end of the episode at *t*. After a long episode of AF this net rate *R*_epi_ will often be negative. To capture the clustering we assume that immediately after the end of an episode the *total* recovery rate *R*(*t*) overshoots and exceeds the natural background *R*_age_(*t*). In order to implement this we introduce an instantaneous boost at the end of an episode: we change the sign of *R*_epi_(*t*) and we allow for an additional factor *B* > 0 in its magnitude: *R*_epi_ → −*B* × *R*_epi_. This is only implemented if *R*_epi_(*t*) < 0 just before the end of the episode, otherwise no reflection or boost are applied. We note that negative rates of *R*_epi_ do not obviously have a direct meaning in isolation. In our model only the *total* recovery rate *R* = *R*_age_ + *R*_epi_, has a direct biological interpretation. This rate is always positive.

The different features of the dynamics are illustrated in Fig 2, where we also show stylised possible time courses of the activation rate and recovery rate.

### Implementation

The model constitutes a continuous-time stochastic process with discrete and continuous variables. During episodes and during times of sinus rhythm the rates *R*(*t*) and *A*(*t*) evolve deterministically according to the dynamics specified by the ordinary differential equations above. The switching dynamics between *S* = 0 and *S* = 1 (SR and AF) is stochastic and governed by these rates. The model can be implemented using a modified Gillespie algorithm [44, 45] to account for the time-dependent rates governing the statistics of the switching events into and out of AF. We emphasize that the stochasticity of the model lies in the waiting time between discrete events (start/end of AF episodes), but that the activation and termination rates follow deterministic equations in-between such events. Mathematical details of the model are discussed in the Supporting Information (S1 File).

### Model parameters

The parameter set used in the present study is listed in Table 1. Where possible, we have estimated parameter values from the literature, as indicated in the table and discussed below.

**Table 1:** The parameter set used in model simulations. Where possible, parameters were estimated from literature; certain parameters were set to reproduce incidence behaviours from epidemiological studies.

Parameter	Description	Value	Unit	Comment/Reference
*A*_0_	Gene. predis. act.	10^-5^	1/yr	Set arbitrarily small
*A*_1_	Max. age/disease act.	2920	1/yr	Paroxysmal AF patients have up to 8 episodes/day [46]
*A*_max_	Max. episode act.	2 × *A*_1_	1/yr	AF doubles VW in tissue [24]
*t*_*c*_	Age/disease transition time	70	yr	Mean incidence rate ([47] table 1)
*t*_*d*_	Age/disease transition width	3	yr	Gives range ±10 years
*α*	Episode act. saturation rate	52	1/yr	AERP stabilises after one week [48]
*β*	Episode act. relaxation rate	182.5	1/yr	AERP recovers after two days [49]
*R*_0_	Peak recovery rate (r.r.) (of new born)	2	1/s	Min possible period of AF is 0.5 sec
*λ*	Nat. degradation rate of r.r.	1.2/50 * log(840)	1/yr	Set so that patient has a 14.4 min episode at age 50
*μ*	Degradation rate of total r.r. in AF	*α*	1/yr	Follows *α*
*ν*	Relaxation rate of epi. r.r. when Healthy	*β*	1/yr	Follows *β*
*B*	Boost factor of r.r. post AF	1	n/a	1: overshoot×1; 0: no o/s

In the following we will occasionally omit units in activation and recovery rates, as they are always understood to be per year. The numerical values of the different components of *A*(*t*) can then be set individually, as shown in Table 1. We focus on investigating effects of natural history and of AF-induced remodelling, and thus set the activation rate due to genetic predisposition *A*_0_ to be very small at 10^−5^. Ageing is a key predictor of AF activation [50], with incidence and prevalence rates increasing with age. For the activation rate due to ageing/co-morbidity, *A*_age_(*t*), we set the maximum value *A*_1_ as 365 × 8 = 2920 on the basis of Hoffmann et al [46] who reported a median of 8 episodes per day in a patient cohort with paroxysmal AF. For activation rate due to AF history *A*_epi_, we cite a recent computational study from Colman et al [24], who predicted that chronic AF doubles the vulnerable time window (in which the atria is susceptible to formation of AF-inducing re-entrant circuits) compared to baseline; we take this to assume that *A*_max_ = 2 × *A*_1_.

To identify the parameters of the sigmoid curve describing increase in activation rate due to ageing, we looked at the incidence rates of AF in several population studies [47, 51–53] and chose parameters *t*_*c*_ = 70 (yr) and *t*_*d*_ = 3 (yr), representing a sigmoid centred at 70 years of age, with a range of approximately 10 years.

The saturation and recovery rates for episode-related activation rate were estimated from a series of experimental studies establishing the existence of AF-induced AF over timescales of days to weeks [11, 14, 49]. They analysed the *atrial effective refractory period* (AERP), a clinical biomarker of atrial vulnerability, over periods of artificially induced AF and looked at changes relative to baseline along with recovery rates post pacing. AERP is known to increase in the right atria with age [54] but shortens when in AF. From these studies, we set *α*, the episode-dependent saturation rate of the activation rate, to be 52 (1/yr), corresponding to a time period of 1 week [48] and *β*, the episode dependent relaxation of the activation rate to 182.5 (1/yr), corresponding to a maximum relaxation period of 2 days [49].

For the parameters governing the recovery function *R*(*t*), we chose to estimate these based on intuition of the AF remodelling process. We set the peak recovery rate *R*_0_ as 2 (1/s), in which we assume the shortest possible duration of an AF episode is 0.5 seconds, and that this is the fastest possible recovery time from AF of a (new born) patient in perfect health. The decay rate of the natural AF recovery function, *λ* was derived on the basis that, from *R*_age_ = 2 (1/s) at *t* = 0, a typical episode of a 50 year old patient would be of the order of 14.4 minutes in duration (1% of a day). This parameter would then be *λ* = log(864 × 2)/50 × 1/*yr*; in our simulations, we approximated this with a slightly larger value of *λ* = 1.2 × log(840)/50 (1/yr).

Finally, for the episode-dependent recovery rate *R*_epi_(*t*), we assume that the degradation rate *μ* follows the saturation rate of episode-dependent activation rate, *α*, and that the relaxation rate *ν* similarly follows the corresponding relaxation, *β* in the activation rate function. For the boost factor *B* (the overshoot above baseline immediately after an episode terminates), we set this to 1, which exactly reflects the decreased amplitude in recovery rate from baseline due to the present episode of AF.

## Results

The primary output of simulations of the model dynamics are patient trajectories. Each run generates a series of AF episodes over several decades; typically we see gradual progression of AF from paroxysmal to permanent. These synthetic data differ from existing clinical AF data: real-world AF ECGs are sampled at several kilohertz over short monitoring periods from hours to months, whilst our data simplifies the ECG in to sequences of binary AF/sinus rhythm episodes. The theoretical resolution of the model is dictated by the shortest episode generated by the model parameters.

We stress that the model parameters used are identical for all simulated patients. Each virtual patient has characteristics identical to the population average. Thus, our model does not capture heterogeneity in those parameters. A systematic analysis of the full parameter space is beyond the scope of the present work, but we highlight, through an initial set of exploratory stochastic simulations, the range of possible trajectories that this model can generate. It should be noted that different runs of the simulation will lead to different sequences of AF/SR episodes for an unchanged parameter set as listed in Table 1. This heterogeneity comes from the inherent randomness of the initiation and termination dynamics in the model.

Results reported below are from simulations of 5000 independent patients. On each simulation run, the patient lives to age 100 years, develops paroxysmal AF at some point and is found to enter permanent AF in their lifetime. This is a simplification of reality, which allows us to analyse the different patient trajectories as a consequence of both long- and short-term remodelling. We will consider different choices of model parameters below, in which some of these assumptions are relaxed. We chose not to consider progression to persistent AF within this study, focussing entirely on paroxysmal and permanent AF.

### Patient trajectories and AF progression

**Fig 3:**
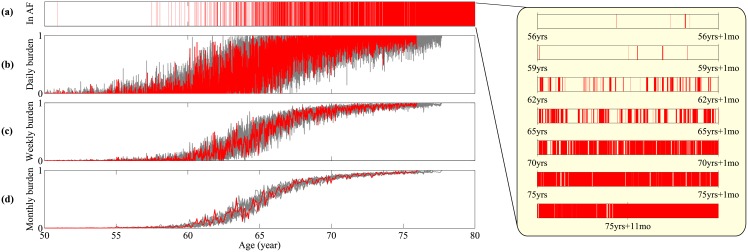
Time series of AF progression. A sample path is visualized by the red line, and from top to bottom: (a) Binary signal whether the patient is in an AF episode, (b) the fraction of the time in AF episodes per day, (c) the fraction of the time in AF episode per week, (d) the fraction of the time in AF episode per month. In (b-d) 9 other sample paths are plotted in grey lines in order to illustrate the distribution induced by intrinsic stochasticity. Inset: the progression of AF episode frequency and duration at time points in the simulation.

Fig 3(a) illustrates a sample time series plotted over a 30-year interval. At any time the patient is either in AF or in sinus rhythm; episodes of AF are highlighted in red. In this example, an initial period of quiescence gives way to a sequence of AF episodes of increasing frequency and duration. As can be seen on the magnification on the right-hand side of the figure, where each time series represents a single month, there are many short and clustered episodes which may not be visible over the longer time-scale view. These eventually become longer in duration until finally the patient remains in AF constantly until death (not shown).

The red (dark) curves in panels (b)-(d) of Fig 3 show the daily, weekly, and monthly AF burden of the patient trajectory shown in panel (a). Following standard conventions [46, 55], burden is here defined as the fraction of time spent in AF during a given observation window. To illustrate the variability of model outcomes panels (b)-(d) depict 9 other sample paths (light grey). AF burden shows a monotonic increasing trend. Naturally the patient-to-patient variance reduces for longer observation windows (monthly versus daily).

Using the parameter set in Table 1, we find that all patients develop paroxysmal AF at some point, and that they eventually enter permanent AF before the endpoint of the simulation at age 100.

**Fig 4:**
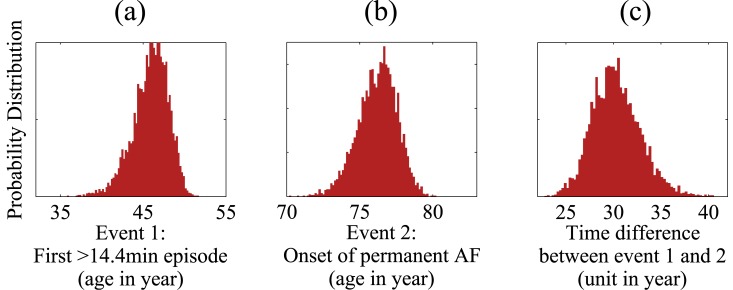
Distributions of age at which paroxysmal AF sets it (panel (a)), age at which permanent AF sets in (panel (b)), and the time elapsed between these two events (panel (c)). Data were generated from 5000 independent simulation runs of the model.

The model then allows us to simulate population statistics for the age at which patients develop paroxysmal AF (event 1) and for the time at which they transition into permanent AF (event 2). These are shown in Fig 4 (panels (a) and (b)), along with statistics of the time that elapses between the onset of paroxysmal and permanent AF (panel (c)). It seems reasonable to assign a minimum threshold which would prompt a patient to present themselves for clinical diagnosis (assuming symptomatic AF). We set this threshold as an AF episode of duration greater than 14.4 minutes (1% of a day). The model results show that the first episode of AF lasting longer than the threshold typically occurs when patients are aged around 44–48 years, which may be perceived as earlier than recorded in the literature [47, 51–53]. Patients subsequently progress to permanent AF over a period of 28–32 years (panel (c)).

### Behaviour after onset of paroxysmal AF

We now turn to a discussion of model outputs in a form that may be clinically relevant. In our model we assume that the starting point of a patient’s clinical trajectory is the time at which they develop paroxysmal AF. Given the intrinsic stochasticity of the model, this time will vary from patient to patient, as indicated in panel (a) of Fig 4.

**Fig 5:**
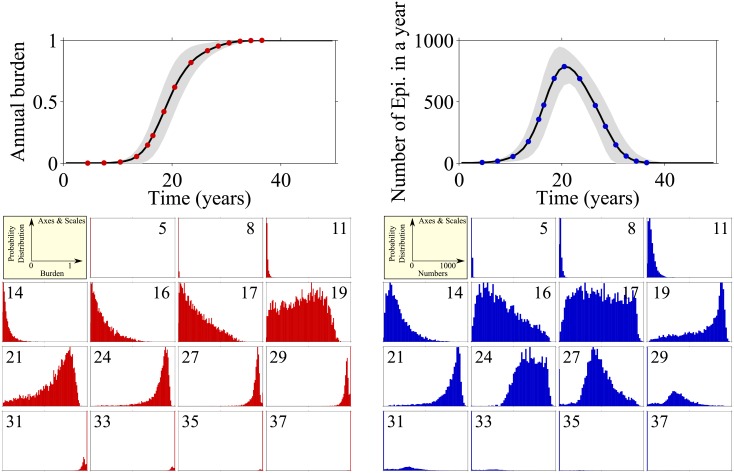
Left: Top panel shows annual AF burden (left) and number of episodes (right) after patient has entered paroxysmal AF. Horizontal axis indicates time elapsed since onset of paroxysmal AF. The mean of 5000 simulation runs is indicated as solid line, grey area shows the standard deviation. Smaller panels show distribution of burdens (left) and numbers of episodes (right) across the patient population at the indicated points in time (years after onset of paroxysmal AF) in a moving window of 12-months duration.

For a single patient we can use the time at which they enter paroxysmal AF as a reference time to follow their future progression. Data are shown in Fig 5; the horizontal axes in the top panels of the figure indicate time after the onset of paroxysmal AF.

In the top left panel of Fig 5, annual AF burden is shown. The burden increases from near zero to one, representing the increase in the proportion of time spent in AF as the condition progresses. Each point on the curve represents the mean burden over 5000 simulated patients.

In the lower-left part of the figure are histograms of AF burden at the 15 time points indicated in the top-left panel. The numbers in the smaller panels refer to the number of years since the onset of paroxysmal AF. At initial times after the onset of paroxysmal AF the burden shows a unimodal distribution, and over the following 17 years the burden increases for some patients while remaining low for others producing a left skewed distribution. Around year 19 there is a shift towards a higher burden and the distribution becomes more uniform, indicating that those patients that previously had low burden are now experiencing the effects of remodelling. By year 21 these effects are evident in even more patients and the distribution becomes right skewed. Finally over years 24–37 all patients experience the effects of remodelling and progress to permanent AF indicated by the distribution tending to a burden of 1.

The right-hand side of Fig 5 shows the total number of episodes per year (top panel) for the same dataset, along with detailed breakdowns of the population statistics in the lower right-hand panels. The number of episodes per year rises sharply and peaks at around 20 years after the onset of paroxysmal AF, and then gradually decreases. This reflects the observation that AF episodes begin intermittently, and become more frequent as structural remodelling increases activation rate and decreases recovery rate. Consequently the episode durations lengthen and the inter-episode times shorten, so the number of episodes is reduced though the time spent in AF (AF burden) increases (left-hand panels of Fig 5).

Initially the number of episodes experienced per year follows a unimodal distribution, with a peak at low numbers. The pace with which AF progresses in different virtual patients varies considerably; some will still have infrequent episodes so a low number of episodes per year, others will have experienced a moderate degree of re-modelling and will have many very frequent episodes. A third group will have experienced substantial structural re-modelling and so they have fewer episodes of longer duration. This leads to the broad distribution at around 17 years after the onset of paroxysmal AF. Eventually all patients will progress to permanent AF and therefore have fewer episodes (19–27 years after threshold). Finally AF is permanent, i.e. there is one continuous episode, and then the centre of the distribution moves towards one single episode per year.

### Behaviour prior to onset of permanent AF

**Fig 6:**
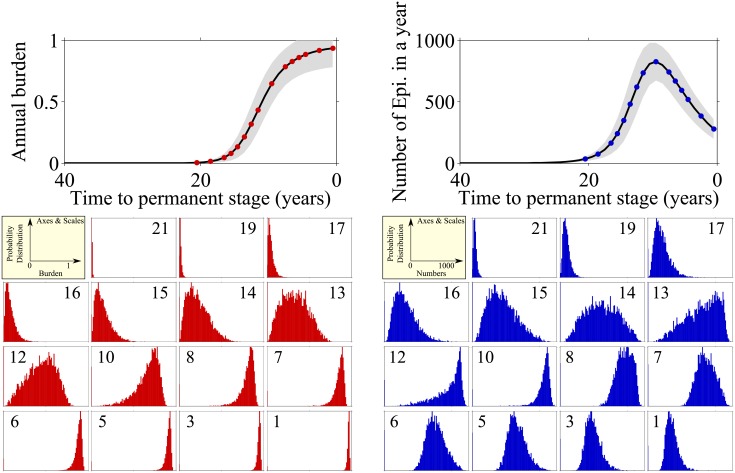
Burden (left) and number of episodes (right) before patient goes into permanent AF. In both panels we present the mean of the random variables (solid black) and mean ± 1 standard deviation (grey area) at the top, and at the bottom we measure the distributions of the random variables at 15 sampled time *t* = [21, 19, 17, 16, 15, 14, 13, 12, 10, 8, 7, 6, 5, 3, 1] years before the permanent episode.

We now change perspective and look at patient progression relative to the point in time at which they develop permanent AF. As discussed, different patients can progress differently after the onset of paroxysmal AF, and there will be considerable variation of the time at which they enter permanent AF. To allow for the identification of potential patterns that can determine progression to permanent AF, we replot the model outputs in Fig 6. Time is now defined relative to the point at which permanent AF sets in, the horizontal axis in the top panels now depict time until the onset of permanent AF. The left-hand panel shows the annual AF burden, for the time leading up to the moment of transition to permanent AF, the panels on the right show data for the number of episodes experienced per year.

From the left-hand panels in Fig 6 we can see that the annual burden begins to increase very slowly until 16 years prior to transition to permanent AF. At that point, patients start to progress at different speeds causing the distribution to become wider (years 14–12 prior to transition). By 10 years before transition the patients are experiencing fairly high burden, which continues to gradually increase until they enter permanent AF.

Examining the right-hand panels in Fig 6 one sees that the number of episodes at 17 years prior to transition has noticeably increased but the corresponding burden (left-hand panel) is still low. This indicates that there are many short episodes—and we know that these are particularly unlikely to be picked up clinically simply due to timing. The number of episodes continues to increase and at 14 years prior to transition the distribution for the number of episodes becomes more unimodal and symmetrical (ignoring a peak at zero episodes, reflecting patients who have not yet entered AF). The distribution shows a wide range in the number of episodes, with the corresponding burden also starting to increase. This illustrates that some people are experiencing the effects of acute AF-induced remodelling, and having many clustered episodes. At 12 years before transition we see that most patients have many episodes, but burden still follows a relatively normal distribution. This indicates that some patients are experiencing the effects of long-term re-modelling and the duration of the individual episodes are increasing, while other patients are only experiencing the effects of short-term re-modelling and although they experience many episodes they are of short duration. From 6 years until the transition to permanent the number of episodes decreases as the duration increases, until eventually the burden is one and the patient remains in AF.

### Model behaviour with different parameters

To demonstrate the ability of our model to capture different patterns of AF progression seen in clinical settings, we now discuss the outcomes for modified sets of model parameters, deliberately focussing on a small number of parameters as an exemplars. Specifically, we independently vary 3 of the 12 parameters listed in Table 1, and compare simulation results against those from the baseline parameter set (see Table 1). We vary the maximum activation rate due to age (*A*_1_), the maximum activation rate due to AF episode-induced remodelling (*A*_max_), and the rate of degradation of the long term recovery rate (*λ*). In addition to the analysis above, we carried out a simple one-at-a-time sensitivity analysis for the 12 parameters of the model, and this data is plotted in Supporting Information (S1 and S2 Figs).

**Fig 7:**
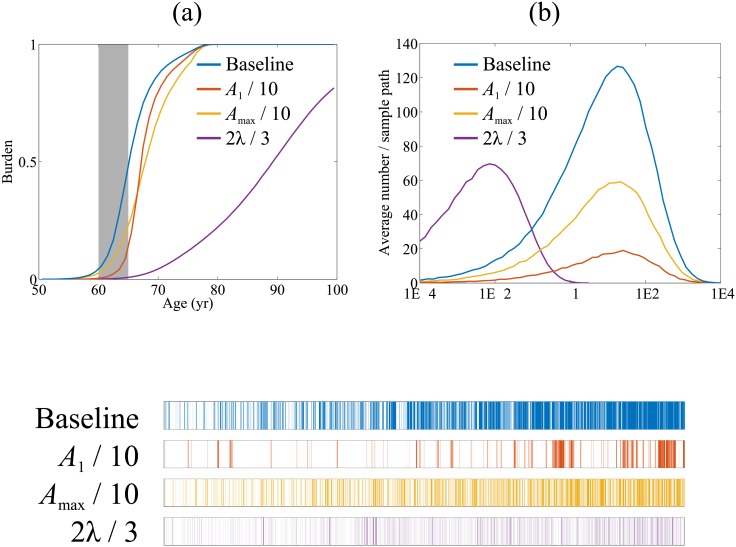
Effects of changing parameters on outputs. The top graphs represent the average over 100 sample paths of (a) the annual AF burden versus absolute age up to a notional age of 100 years, and (b) number of episodes versus episode duration over the absolute age 60–65. (c) is a visualisation of a single sample path from each parameter variant over the same 60–65 period a (b). Additional sample paths are listed in Supporting Information (S3 Fig).

Fig 7 shows simulation results from the baseline parameter set (blue), reducing *A*_1_ (red) by a factor of 10 and, independently, reducing *A*_max_ (yellow) by a factor of 10, and, again independently, reducing *λ* (purple) by a third. The choice to decrease the parameters was for ease of presentation; increasing the parameters had the converse effect to the presented graphs (data not shown). For each variation we generated 100 independent sample paths.

In panel (a) we focus on the time behaviour of AF burden per year for the different parameters sets. To simulate model outputs in a clinical setting, we analyse episodes occurring over a 5-year monitoring period between age 60 to 65 for each of the parameter variants. The distributions of episode durations in this monitoring window are shown in Fig 7(b), where we plot histograms indicating the average number of episodes of a given duration per patient trajectory for each of the parameter sets. Panel (c) shows representative sample paths obtained from simulations with each of the four parameter sets.

**Baseline:** With the baseline parameter set, burden increases in a sigmoidal fashion between the age of 60 and 80, the average AF burden reaches one at around age 80. All patients have progressed to permanent AF by this age. An alternative perspective is given by the representative baseline sample path shown in panel (c) (upper most plot), which indicates a gradual reduction in intervals between episodes of AF over the 5-year period.

**Reduced maximum vulnerabilities due to age and AF episode-induced remodelling (*A*_1_ or *A*_max_):** The effect of reducing either *A*_1_ or *A*_max_ is to delay the initial onset of progression to permanent AF, and this can be seen in Fig 7(a). However, the average annual AF burden again tends to 1.0, similar to baseline, at a later age of about 80 years. The distributions for reduced *A*_1_ and *A*_max_ shown in panel Fig 7(b) indicate a lower number of AF episodes relative to baseline model without significantly altering episode durations.

The representative sample paths in panel (c) show qualitative differences between simulations with reduced *A*_1_ (maximum activation rate due to age) and *A*_max_ (maximum activation rate due to AF-induced remodelling). With reduced *A*_1_ we observe marked clusters of episodes in the five-year observation window (second time series in panel (c)). These are not seen in the representative sample path for simulations with either reduced *A*_max_ or baseline parameters (see Supporting Information S3 Fig for additional sample paths). A possible explanation for this observation is that when age related activation rate is reduced, other components of activation rate including the boost shown in Fig 2(a) act to increase the rate at which new episodes of AF occur immediately following termination, and so contribute to clustering. Reducing *A*_max_ did not lead to a qualitative change in the pattern of AF episodes.

**Slowing down the age-related decline of recovery rate:** Reducing the model parameter *λ* has a more significant impact on AF progression, delaying the both onset of AF and progression. The final AF burden at the end of simulations (age 100 years) is no longer equal to one (Fig 7(a)), so not all patients progress to permanent AF. The total number of episodes observed per patient is significantly lower than at baseline, and the median episode duration is now an order of magnitude lower (Fig 7(b)). This behaviour results from the higher recovery rate compared to baseline, which acts to increase the likelihood of an AF episode terminating. The representative sample path for reduced *λ* shown in Fig 7(c) shows a large number of short episodes.

In presenting the results for changed parameters we have partly relied on data averaged over ensembles of simulation runs (e.g. the data shown in panels (a) and (b)), but also on the sample paths shown in panel (c). We recognise that results from a single path may not be representative and thus we show trajectories of 20 sample paths for each parameter set in the Supporting Information (S3 Fig).

## Discussion

### Summary of the model and its outcomes

In this study, we have introduced a stochastic, individual-based model of the initiation, maintenance and long-term progression of AF. The model is a quantitative tool, which represents the conceptual model of AF progression shown in Fig 1 [4, 36]. Our model is based on initiation and termination rates for AF episodes that are dynamic and depend on past AF history. Parameters are estimated from the literature or by intuition when suitable data could not be found. The simulation of a single patient returns a time series data of AF episodes. Evaluating an ensemble of sample paths for a given parameter set enables a patient population to be simulated, and results in a probability distribution of characteristic outputs. These include AF metrics such as annual burden or number of episodes in a year. We use these probability distributions to hypothesise how a patient might progress to permanent AF after being diagnosed with paroxysmal AF, and to analyse behaviour prior to onset of permanent AF. We consider the effect of varying some of the model parameters, and determine how this would alter the temporal progression of AF within the model. Such an analysis shows that some features of the basic model are retained, but that quantitatively different AF behaviour can be generated. For example, different choices of the model parameters leads to different probabilities with which virtual patients progress to paroxysmal AF, or to different progression rates between the different stages of AF.

This approach has a number of potential applications, which we discuss below.

### Potential applications for the model

#### A quantitative model to link mechanistic and population-level descriptions of AF

Our approach enables the parameters and interactions that are important for controlling progression of AF to be explored, and formalises ideas such as “AF begets AF” in a way that is more difficult with a conceptual model alone. Based on the success of similar approaches in other fields (e.g. epidemiology, cancer modelling or gene regulation) we believe that stylised models such as the one we discuss here can develop into useful practical tools. The model and the associated parameters make a novel contribution to discussion in this field, by providing a framework to examine AF initiation, maintenance and progression. There is the potential to link with biophysical models addressing cellular and organ-level processes of AF on the one hand, and with population level models on the other, which act on large time scales of decades. Within the larger picture of “verification, validation and uncertainty quantification” [56] our approach has the potential to reduce our overall uncertainty about how AF progresses, and has the potential to provide a quantitative way to combine, compare and verify experimental, clinical and epidemiological data.

#### Correlating AF mechanisms with progression of AF

Our present model is a simplified representation of disease progression in patients, and so it does not explicitly take into account all factors that may impact the rate of AF progression. In particular the model does not describe any sort of intervention at this stage. It does, however, allow the opportunity to vary parameters, individually or as sets. This enhances our understanding of the contributions and interplay of each of the parameters and mechanisms. By systematically varying model parameters we can study how the different components of the model relate to initiation and termination of an AF episode at different stages of a patient’s life. Ultimately this will allow one to correlate different initiation and termination mechanisms to long-term AF progression.

In this study, we have made first steps in this direction, based on the initial model we have presented. Varying the model parameters *A*_1_ and *A*_max_ for example (maximum activation rate due to age and AF episode-induced remodelling respectively) adjusts the relative impact of long-term (age-based) versus short-term (episode based) activation rate. These are associated with structural and functional remodelling respectively. By making the long-term activation rate the dominant effect, AF episodes in our model show a more gradual increase in both frequency and duration over the observed time window. In contrast, when episode-based activation rate is dominant, the frequency of episodes over a fixed time window is strongly history-dependent which would lead to clustering events.

At this stage these are outcomes of the model only, and we make no claim of immediate relevance to clinical practice. However, further refinements of the model are possible. For example it would support the general approach and setup of our model if outputs and interrelations between model components (such as the ones described above) can, in some form, be identified from clinical settings. This would be useful even at a *qualitative* level. If this is the case, there are further refinements and additions to make the model more detailed, and more accurate, both quantitatively and qualitatively. Similarly, if model predictions are not borne out in real-world patient populations, modifications of the model are indicated. Either way, and even in the absence of *quantitative* parametrisation and validation, we feel there is scope for further interaction and development.

For the purposes of our present model, we class a patient as being in persistent AF once they have their first seven-day episode, regardless of whether this is followed by many short-term self-terminating episodes or not. This differs from the clinical classification where a cardioversion may be attempted prior to a 7-day event, resulting in the patient to be re-classed as being in *persistent AF*. This means that the results from our model may over-estimate the time of transition from paroxysmal to persistent AF—though this is not explicitly reported in this paper. Similarly, for the purposes of our model the patient is deemed to be in *permanent AF* once they reach a constant state of AF, and this differs from in a clinical setting where interventions have failed, causing the model to again over-estimate the time to transition from persistent to permanent AF. This demonstrates a limitation in using the standard definitions to describe AF progression which are based on a combination of clinical decisions (e.g. no further attempts are made to restore SR), and physiological factors (i.e. reported patterns of AF events) in calibrating the model.

#### The model as a tool to evaluate clinical measures of AF progression

In order to be able to describe AF progression, one needs some way of quantifying the status of any one patient, and their progression from one stage of AF to the next. In-line with existing literature [55] we have used AF burden (the time spent in AF as a proportion of time monitored) as an indicator. Accurately determining the AF burden of a real patient can be problematic though, as AF is an intermittent condition. As a consequence the burden measured during a period of monitoring is related to the choice and duration of that monitoring window. Burden on its own is also insufficient to describe clustering of episodes. Charitos et al [55, 57] have recently devised a measure of “AF density” which provides a useful measure describing the dispersion of events over a set time period. At the same time this new indicator carries other limitations centred around the length of the monitoring period [58]. In future, improved versions of the model one may hence think of additional measures of AF status and progression. In particular models of the type we have discussed here can be of use in assessing these different measures and in identifying their relative advantages and potential pitfalls. In order to do this, fully validated models are not required, and simple stylised models like ours may in fact be preferable, as large ensembles of samples can be generated easily. At the same time the time series we have shown demonstrate that the model is able to sample different regimes of episode clustering. This mimics the different progression patterns seen in patients—some quickly progress to a state of permanent AF, others have sporadic periods of AF then long periods of without AF. Our model provides an opportunity to test measures of clustering on surrogate time series in different scenarios.

Ultimately such measures can then be applied to clinical data. This will become more relevant as implanted and trans-dermal monitors become more common and memory capacities increase. Combined with remote access to recordings, this will enable more detailed and longer monitoring data to become viable. Algorithms that monitor events and measure progression in order to aid on-going management of the condition will need to correctly classify the condition of the patient. The model may provide the ability to generate training data for these algorithms in a non-clinical environment, as well as to measure the robustness of potential measures of progression.

#### Capturing the effects of intervention

Assuming further refinements, models such as the one we have introduced have the potential to capture the impact of interventions on progression in very general terms. For example one may think of linking the mechanism drug action to features of the model (e.g. *Ca*^2+^ channel blockade effects on substrate vulnerability and triggering). However, more work would be needed to calibrate the effects of the interventions and the impact on the model parameters.

### Model validation

The initial model has been used to formalise key ideas and mechanisms of AF progression into a framework for simulations and further discussion. The process of model formulation raised several questions about the values we should choose for the model parameters as well as how the model could be tested and evaluated against measurements of AF progression in patient populations. Some of the model parameters, for example *A*_1_ and *A*_max_, could be selected based on values from the literature (Table 1). However, other model parameters, for example *A*_0_ and *β*, could not and so were estimated. While our model reproduces the progression of AF that we would expect to see based on the conceptual model shown in Fig 1, a more formal evaluation is problematic because it would require monitoring of AF patients over an extended period lasting decades. AF is typically diagnosed only once it becomes symptomatic, and so information about early episodes of paroxysmal AF is very difficult to obtain.

## Conclusion

In this paper we have presented a proof-of-concept stochastic model that simulates AF episodes and AF progression based on patient-specific parameters. The model is deliberately stylised and basic. It is designed to allow systematic exploration of the parameter space and to contribute to the discussion of (i) the key mechanisms determining the long-term progression of AF, (ii) the interplay and relative importance of these mechanisms, (iii) the choice of model parameters and of the initial conditions. Iterated discussion of these and qualitative (or at later stages, quantitative) comparison against what is seen in clinical progression will allow for further refinements of the model. We have shown that the model is capable of producing different patterns of AF progression through sample parameter sets, and we believe the model has the potential to correlate parameters describing different mechanisms contributing to AF with long-term progression. We make no claim that our model is validated at this point, and neither is it intended to make quantitative predictions for real-world patient cohorts. It is a toy model, designed to make progress towards linking mechanistic aspects of AF at cellular level and on short time scales with features of AF progression on long time scales and at population level. Establishing such a link on a firm and quantitative basis will require a research programme of iterated tests against clinical observations and refinements of the model. Initially, we expect, this will be at a qualitative level, but successively becoming more quantitative. Nevertheless, our initial model highlights the need for high quality, long-term and individual quantitative data on the progression of AF for use in future models.

If such a programme succeeds one may hope that a valuable simulation tool emerges with which to predict the progression of AF. These simulations would operate at cohort-level and at patient level, starting from specific morphological characteristics of individual patients at the mechanistic level, and delivering output up to the scales of populations. A tool of this type would also be of interest for assessing the prospects of success of different modes of intervention in heterogeneous populations, and for individual patients.

## Supporting Information

S1 Fig Time series visualisations of AF episodes between ages 50–80, varying each of the parameters of Table 1 one-at-a-time. Parameters 1–6. All parameters except *t*_*c*_ and *λ* were rescaled by a factor of 10 and 0.1; *t*_*c*_ was shifted by -10 and 10 years, whilst *λ* was rescaled to 0.9× and 1.1× parameter values. (a) and (b): red visualisation denotes a single sample path, in grey are 9 other sample paths. Top panel—AF time series, second panel—daily burden, third panel, weekly burden, bottom panel, monthly burden. (c): average annual burden between age 50–80, from 100 sample paths.
(PDF)

S2 Fig Time series visualisations of AF episodes between ages 50–80, varying each of the parameters of Table 1 one-at-a-time. Parameters 7–12. All parameters except *t*_*c*_ and *λ* were rescaled by a factor of 10 and 0.1; *t*_*c*_ was shifted by -10 and 10 years, whilst *λ* was rescaled to 0.9× and 1.1× parameter values. (a) and (b): red visualisation denotes a single sample path, in grey are 9 other sample paths. Top panel—AF time series, second panel—daily burden, third panel, weekly burden, bottom panel, monthly burden. (c): average annual burden between age 50–80, from 100 sample paths.
(PDF)

S3 Fig 20 time series visualisations of AF episodes during the observation window 60–65 years for the four parameter sets in Fig 7. (a): baseline set from Table 1 (blue), (b) *A*_1_ = 292, other parameters unchanged (red) (c): *A*_max_ = 584, other parameters unchanged (yellow), (d): *λ* = 1.2/75 * log(840), other parameters unchanged (purple).
(PDF)

S1 File Supporting Information of A Stochastic Individual-based Model of the Progression of Atrial Fibrillation in Individuals and Populations. Supporting information accompanies this article.
(PDF)
