# Supplementary figures and images for: A Stochastic Individual-Based Model of the Progression of Atrial Fibrillation in Individuals and Populations
Source: PLoS One. 2016 Apr 12;11(4):e0152349. doi: 10.1371/journal.pone.0152349 (PMC4829251; doi:10.1371/journal.pone.0152349)

(a)

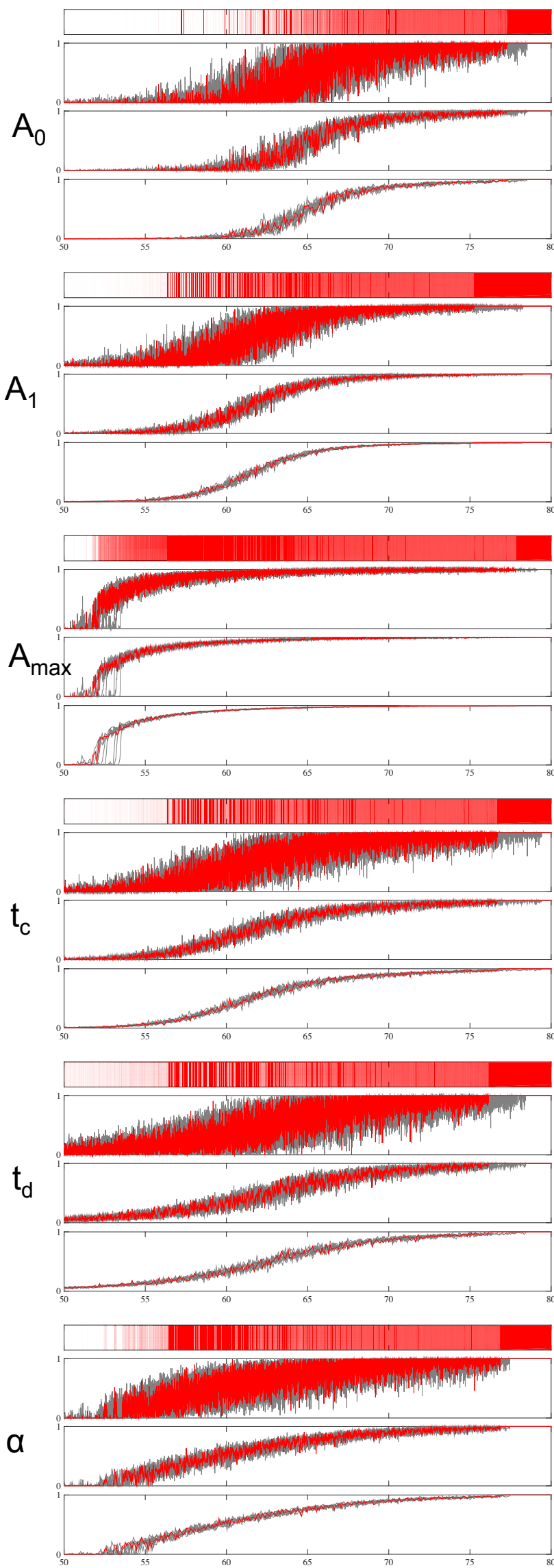

(b)

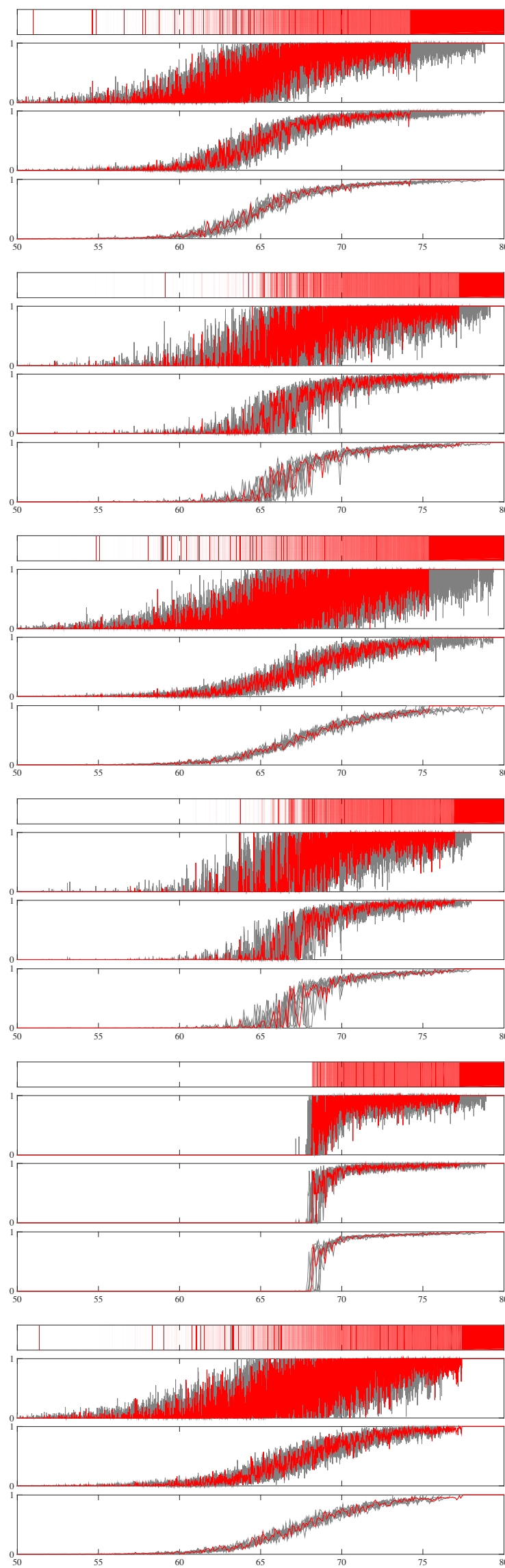

(c)

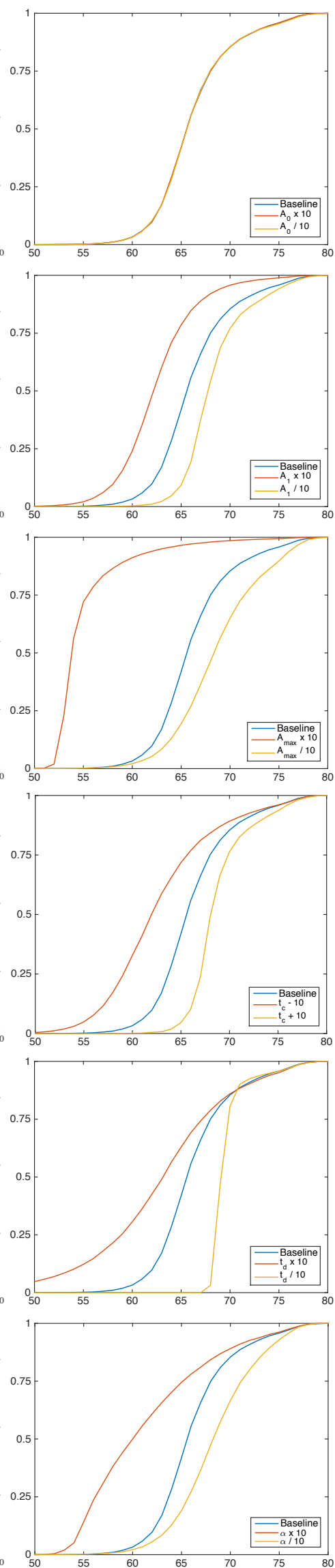

Supplement: S1 Fig — All parameters except tc and λ were rescaled by a factor of 10 and 0.1; tc was shifted by -10 and 10 years, whilst λ was rescaled to 0.9× and 1.1× parameter values. (a) and (b): red visualisation denotes a single sample path, in grey are 9 other sample paths. Top panel—AF time series, second panel—daily burden, third panel, weekly burden, bottom panel, monthly burden. (c): average annual burden between age 50–80, from 100 sample paths. (PDF) [file pone.0152349.s001.pdf]

(a)

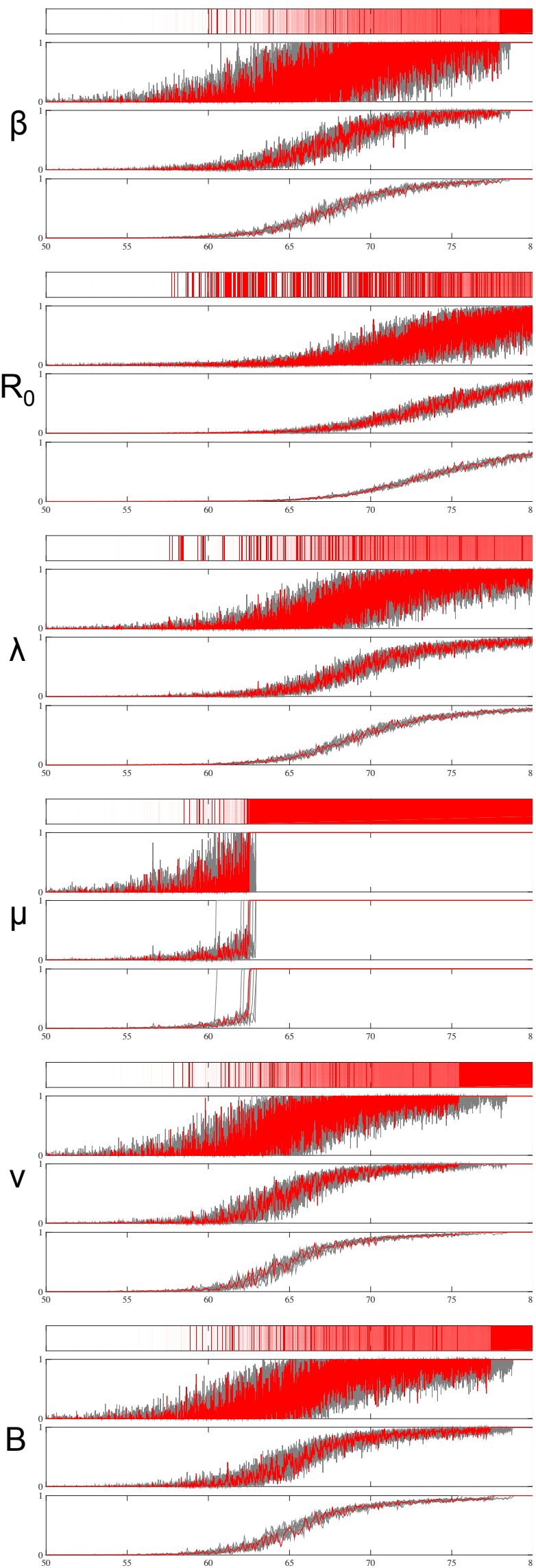

(b)

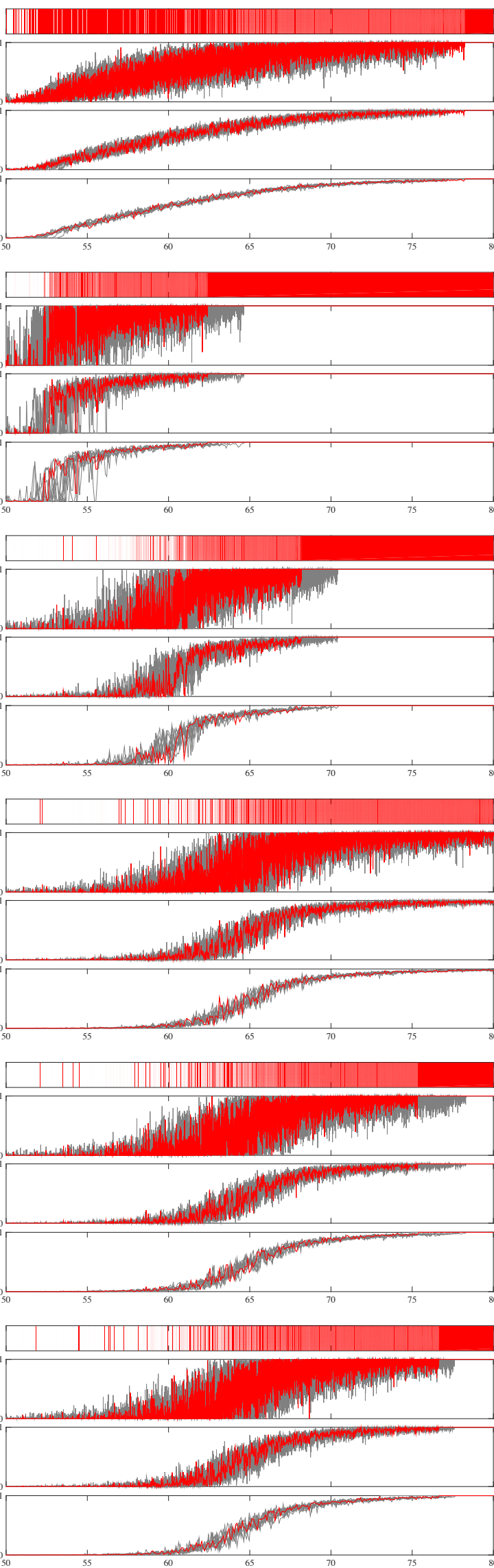

(c)

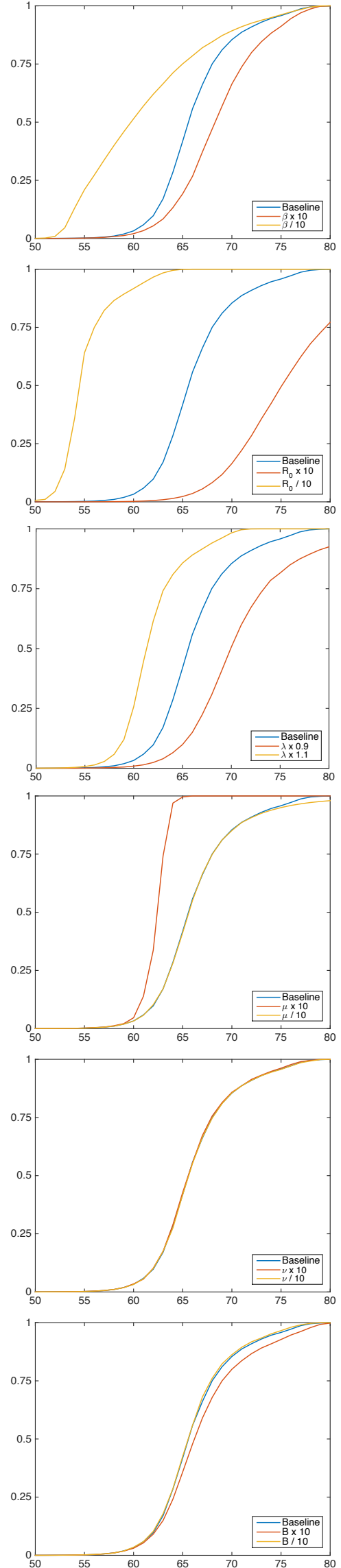

Supplement: S2 Fig — All parameters except tc and λ were rescaled by a factor of 10 and 0.1; tc was shifted by -10 and 10 years, whilst λ was rescaled to 0.9× and 1.1× parameter values. (a) and (b): red visualisation denotes a single sample path, in grey are 9 other sample paths. Top panel—AF time series, second panel—daily burden, third panel, weekly burden, bottom panel, monthly burden. (c): average annual burden between age 50–80, from 100 sample paths. (PDF) [file pone.0152349.s002.pdf]

(a)

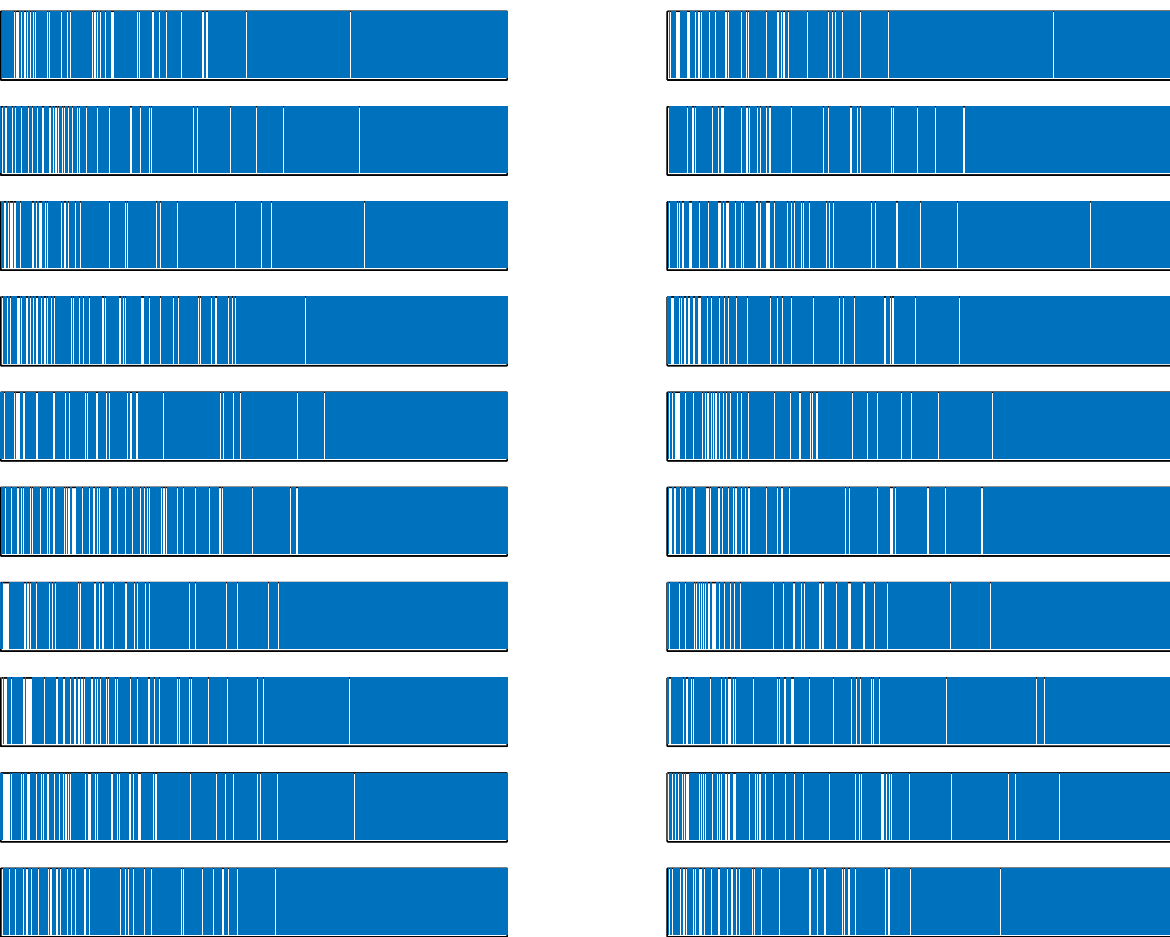

(b)

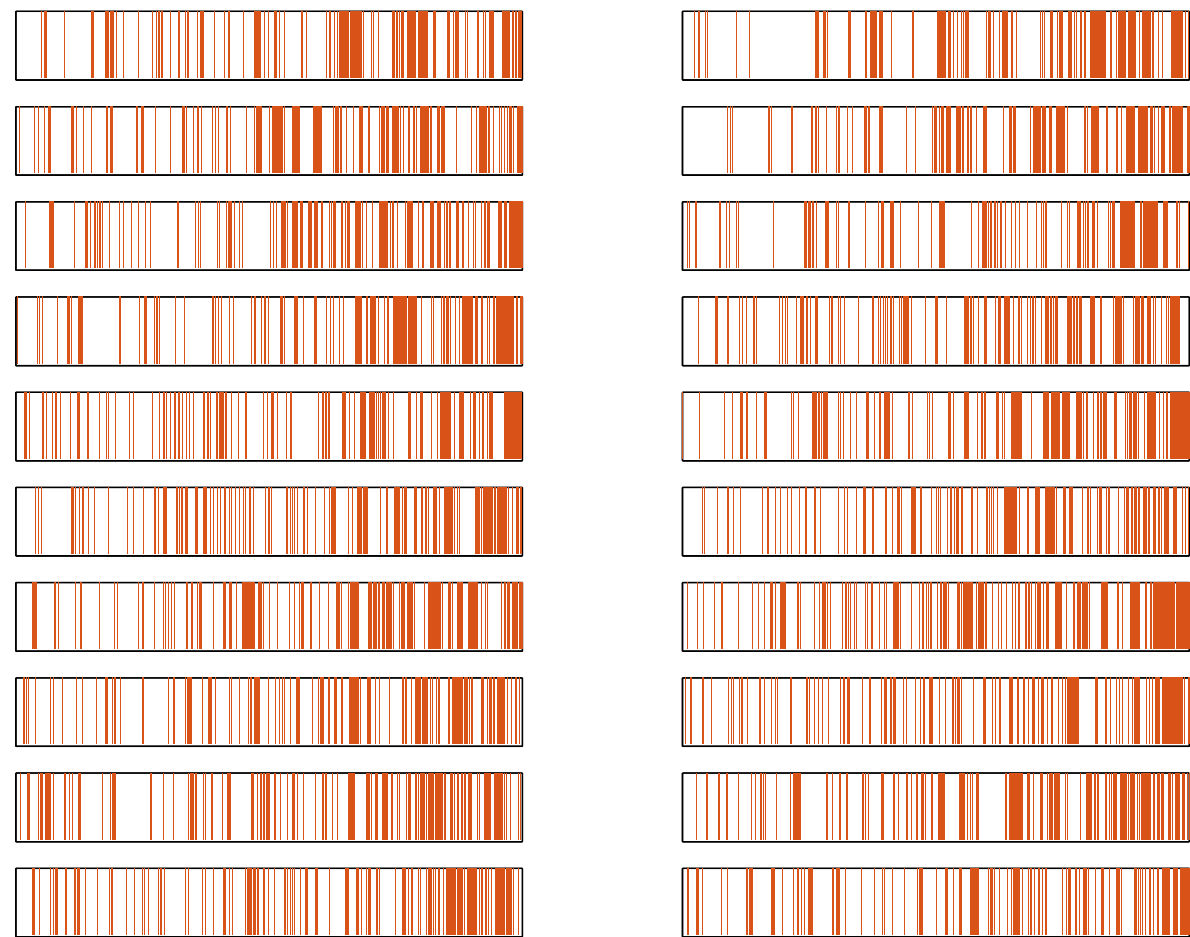

(c)

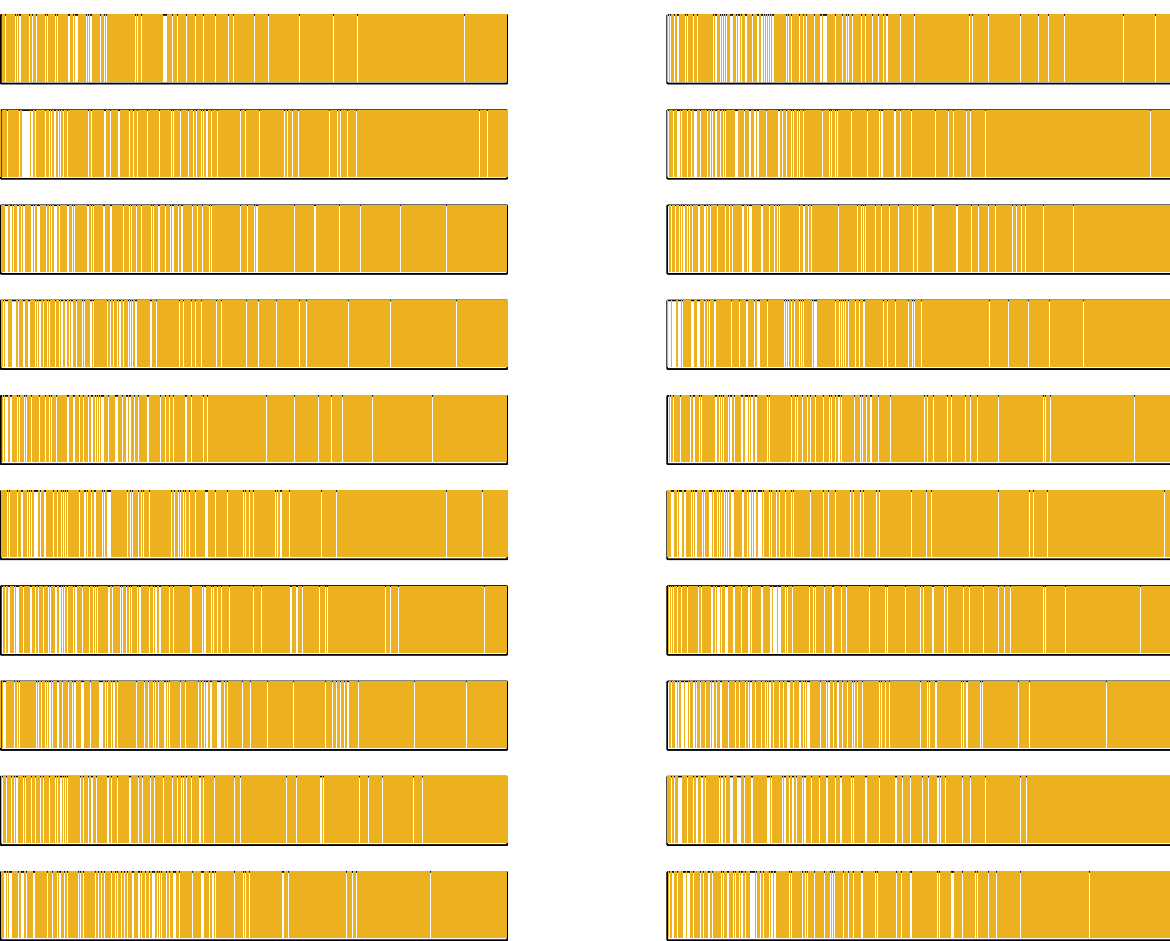

(d)

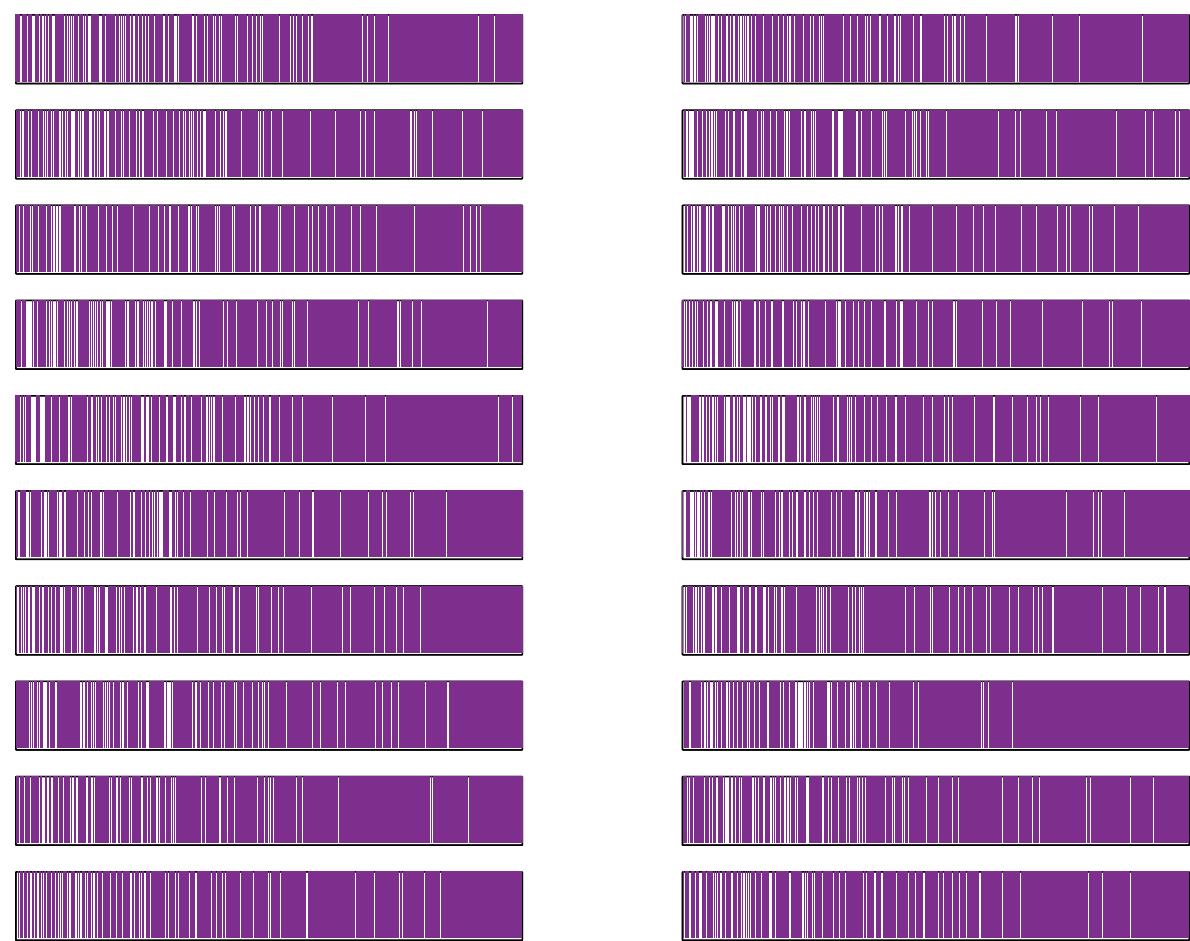

Supplement: S3 Fig — (a): baseline set from Table 1 (blue), (b) A1 = 292, other parameters unchanged (red) (c): Amax = 584, other parameters unchanged (yellow), (d): λ = 1.2/75 * log(840), other parameters unchanged (purple). (PDF) [file pone.0152349.s003.pdf]
