# Supplementary material for: A Stochastic Individual-Based Model of the Progression of Atrial Fibrillation in Individuals and Populations
Source: PLoS One. 2016 Apr 12;11(4):e0152349. doi: 10.1371/journal.pone.0152349 (PMC4829251; doi:10.1371/journal.pone.0152349)
Supplement: S1 File — Supporting information accompanies this article. (PDF) [file pone.0152349.s004.pdf]

Supplementary Information of A Stochastic Individual-based Model of the Progression of Atrial Fibrillation in Individuals and Populations

Eugene TY Chang,[1] Yen Ting Lin,[2] Tobias Galla,[2] Richard H Clayton,[1] and Julie Eatock[3]

[1] *Insigneo Institute for in-silico Medicine and Department of Computer Science, University of Sheffield, Sheffield, S1 4DP, UK*
[2] *Theoretical Physics, School of Physics and Astronomy, The University of Manchester, Manchester M13 9PL, UK*
[3] *Department of Computer Science, Brunel University London, Uxbridge, UB8 3PH, UK*

(Dated: March 17, 2016)

Survival Functions and Random Waiting Time Generation

The transition rates evolve deterministically over time conditional on the current state of the subject. A simple calculation shows that for a given rate, $r(t)$, the random waiting time T for such an event to occur at time t_0 satisfies

$$\mathbb{P}\left\{T > t\right\} = \exp\left(-\int_{t_0}^{t_0+t} r\left(t'\right) dt'\right). \tag{S1}$$

We refer to (S1) as the "survival function" of the state, since it predicts the probability that an event does not occur until time $t_0 + t$. It is noted that when $r(t)$ is constant, the survival function is exponential, as the random waiting time will be exponentially distributed, but in our processes with a changing $r(t)$ the waiting time distributions are in general not exponentially distributed.

For example, when the transition rate is a constant $r(t) = a$, the survival function is

$$S_{\text{con}}(t; a, t_0) = e^{-at}. \tag{S2}$$

For a exponentially decaying rate $r(t; a, b) = a\exp(-bt)$, the survival function is

$$S_{\text{exp}}(t; a, b, t_0) = \exp\left[\frac{-a}{b}\left(e^{-bt_0} - e^{-bt}\right)\right]. \tag{S3}$$

Finally, for the sigmoid function $r(t) = a/(1 + \exp[-b(t-c)]$, the survival function is

$$S_{\text{sig}}(t; a, b, c, t_0) = \exp\left[\frac{-a}{b}\log\left(\frac{e^{bt} + e^{bc}}{e^{bt_0} + e^{bc}}\right)\right]. \tag{S4}$$

From (S2) to (S4) we can derive the distribution of the waiting-times to the next switching event. It is elementary to show that, the waiting times to the next transition event are

$$\begin{aligned}
T_{\text{con}}(a) &= -\log(U)/a, \\
T_{\text{exp}}(a, b, t_0) &= \begin{cases} \frac{-1}{b}\log\left[e^{-bt_0} + \frac{b}{a}\log(U)\right] - t_0, & \text{if } U > \exp\left[\frac{-a}{b}\left(e^{-bt_0}\right)\right] \\ \infty, & \text{else.} \end{cases} \\
T_{\text{sig}}(a, b, c, t_0) &= \frac{1}{b}\log\left[\left(e^{bt_0} + e^{bc}\right)U^{-\frac{b}{a}} - e^{bc}\right] - t_0
\end{aligned} \tag{S5}$$

where $U \sim \text{Unif}(0, 1)$ is a random number uniformly distributed in $(0, 1)$. With (S5), we can construct Monte Carlo simulations to generate sample paths of AF progression.

As a specific example, the rate at which an individual moves from being in sinus rhythm to being in AF is a sum of three rates

$$\text{Activation rate:} A(t) = A_0 + A_{\text{age}}(t) + A_{\text{epi}}, \tag{S6}$$

and it suggests that the waiting time distribution involves three independently and randomly distributed waiting times, each of which can be generated by Eq. (S5).

The outline of the kinetic Monte Carlo simulation is sketched as the following. For each sample path, the simulation begins with patient's age = 0 and in sinus rhythm state. Three random waiting times are generated according to (S6) using formulae (S5). The physical time is advanced by the minimum of the three generated waiting times, at which time the patient's state is switched to the AF state. A similar procedure is carried out to advance time to the switching event from AF state back to sinus rhythm. The procedure is repeated until the patient is 100 years old, and the progression of many (5000) patients with identical parameters are stored for statistical analysis.

Sensitivity Analysis of Model Parameters

In S1 Fig and S2 Fig, we display sample paths over ages 50-80 for a simple one-at-a-time analysis of the 12 parameters in the model, listed in Table 1. In this analysis, we scaled each parameter by a factor of 10 and 0.1 in turn, except for t_c (shifted by -10 and 10 years) and λ (scaled to $0.9\times$ and $1.1\times$ baseline).

Columns (a) and (b) display a single sample path highlighted in red for each parameter with the time series plus daily, weekly and monthly burden, overlaid on 9 other sample paths in grey. Column (c) shows the average annual burden over the same time period over 100 sample paths. As shown in the figures, many of the changes had no significant qualitative effect on AF progression from the baseline parameter set (S3 Fig other than shifting the progression along the time axis. However, for certain parameter choices ($A_{\max}$ (a), t_d (b), R_0 (a) and (b), λ (b) and μ (a)), we obtain a variety of AF progressions which remain in paroxysmal or persistent AF, or rapidly progress in to permanent AF shortly after initial onset. This is in line with existing knowledge that patients experience different progression pathways, with some remaining in paroxysmal AF over their lifetime, and some progression to persistent or permanent AF shortly after initial AF diagnosis.

This simplistic one-at-a-time analysis is a very preliminary study in to the parameter space of the model, but already suggests that the model has the capacity to generate, via parameter modulation, a large variety of synthetic AF progression data which could be used to probe hypotheses regarding the variability of AF progression observed clinically, or be used as a tool to simulate effects of therapeutic targets on subsequent progression.

Sample paths for different choices of model parameters

In S3 Fig, we display 20 sample paths from our stochastic simulations showing typical time series when the patients are between 60 and 65 years old.
